# AI-Based Personalized Therapy With Clinical Intelligence and Radiomics (SPOILS) for Patients With Low Back Pain: Prospective Observational Study

**DOI:** 10.2196/83322

**Published:** 2026-03-11

**Authors:** Purushottam Kumar, Suyash Singh, Bunil Kumar Balabantaray

**Affiliations:** 1 Department of Neurosurgery, All India Institute of Medical Sciences, Raebareli, Dalmau Road, Munshiganj, Raebareli, Uttar Pradesh, 229405, India, 91 6393627740; 2 Department of Computer Science and Engineering, National Institute of Technology, Raipur, Raipur, Raipur, India

**Keywords:** lumbar spondylosis, personalized treatment, clinical intelligence and radiomics, lumbar spondylosis diagnosis and treatment, spine segmentation, degenerative disease, artificial intelligence, AI

## Abstract

**Background:**

Low back pain (LBP) is a leading cause of disability worldwide, affecting people of all ages while showing increasing prevalence among younger demographics. Patients may present with different symptoms and treatment responses despite identical magnetic resonance imaging results, making it difficult to determine whether surgical and medical interventions are appropriate.

**Objective:**

This study aimed to develop SPOILS (Software to Predict Outcome in Lumbar Spondylosis), an artificial intelligence–based decision support tool that merges clinical intelligence and radiomics to generate customized therapy plans for patients with LBP.

**Methods:**

The SPOILS system used deep learning models to perform automated segmentation, enabling the extraction of geometrical parameters, including disk height, disk width, vertebrae height, vertebrae width, canal diameter, disk height index, signal intensity, and disk volume. A labeled dataset was created using expert-verified Pfirrmann and spondylosis severity gradings to address the clinical issues stemming from manual grading variability and subjectivity. Machine learning algorithms were used with this combined dataset to predict outcomes and recommend personalized treatment plans.

**Results:**

The DeepLabV3+ segmentation model with a ResNet50 encoder achieved 95.5% accuracy, which increased to 98.7% after 8-fold cross-validation and simultaneously improved precision (96.95%), recall (97.1%), Dice coefficient (96.9%), and intersection over union (IoU; 94.8%). The convolutional neural network with MobileNetV2 achieved 97.84% accuracy and 96.76% IoU for spondylosis severity prediction after cross-validation. The Gradient Boost classifier demonstrated the best results with geometrical data by achieving 91.65% accuracy and 84.59% IoU.

**Conclusions:**

SPOILS introduced an innovative method to customize LBP treatment through the combination of artificial intelligence technology with radiological data and clinical expertise.

## Introduction

Low back pain (LBP) is a growing global health concern [1], and there are no clear recommendations regarding whether to pursue surgical or medical management. It is one of the most common chronic diseases worldwide, with an increasing propensity to affect younger age groups [2,3]. Similar-looking magnetic resonance imaging (MRI) findings combined with different subjective pain perceptions may lead to differences in clinical opinion [4]. Moreover, surgical options range from simple epidural injection to interbody fixation. LBP is the leading cause of years lived with disability, accounting for 7.41% of the total years lived with disability [5]. In 2020, there were more than half a billion prevalent cases of LBP globally, and projections indicate that this number will exceed 800 million by 2050. In this era of evidence-based medicine, we blended artificial intelligence (AI) with clinical intelligence to develop a model that uses radiological, clinical, and subjective data to predict the best possible treatment as “individualized therapy,” SPOILS (Software to Predict Outcome in Lumbar Spondylosis). As a standard clinical practice, “low back pain” is initially differentiated into mechanical or neuropathic [6]. Mechanical pain usually responds to rest, ice, and physiotherapy [7], but neuropathic pain requires further radiological investigations. Depending upon radiological features, such as canal diameter, disk extrusion, nerve compression, and foramen stenosis, neurological status, and pain severity, clinicians usually decide on the further management course. In our study, we used all these data and developed predictive analysis software to provide patients with an indication of the treatment they may be prescribed. Additionally, our objective in developing the prediction software (SPOILS) was to provide an objective adjunct that supports evidence-based practice and clinical decision-making of clinicians. Radiomics, when used as an AI tool, is a technique that leverages AI algorithms to extract large amounts of quantitative features from medical images, allowing for detailed analysis of tissue characteristics beyond what can be seen visually. These features can be used to improve diagnosis, prognosis, and treatment planning for various diseases, particularly cancers, by providing more precise information about tumor biology and behavior. These features are used to develop radiomic models or signatures that aid in interpreting various clinical phenotypes, such as patient genotyping, treatment efficacy, and clinical outcomes. Our study focused on the development of a predictive model for lumbar spine spondylosis treatment using a fully automated quantitative analysis system. The automated system accurately identified and quantified spinal structures, demonstrating successful segmentation and disk volume extraction. These models aim to support clinical decisions regarding surgical interventions for patients with spondylosis.

## Methods

### Ethical Considerations

Ethics approval (2023‐6-EMP-4) was obtained from the institutional ethical committee of All India Institute of Medical Sciences (AIIMS), Raebareli, India, on May 23, 2023. The retrospective nature of the data and the use of deidentified magnetic resonance (MR) images ensured patient confidentiality and adherence to ethical standards. Written informed consent was obtained from participants as well.

### Study Design

**Figure 1.**
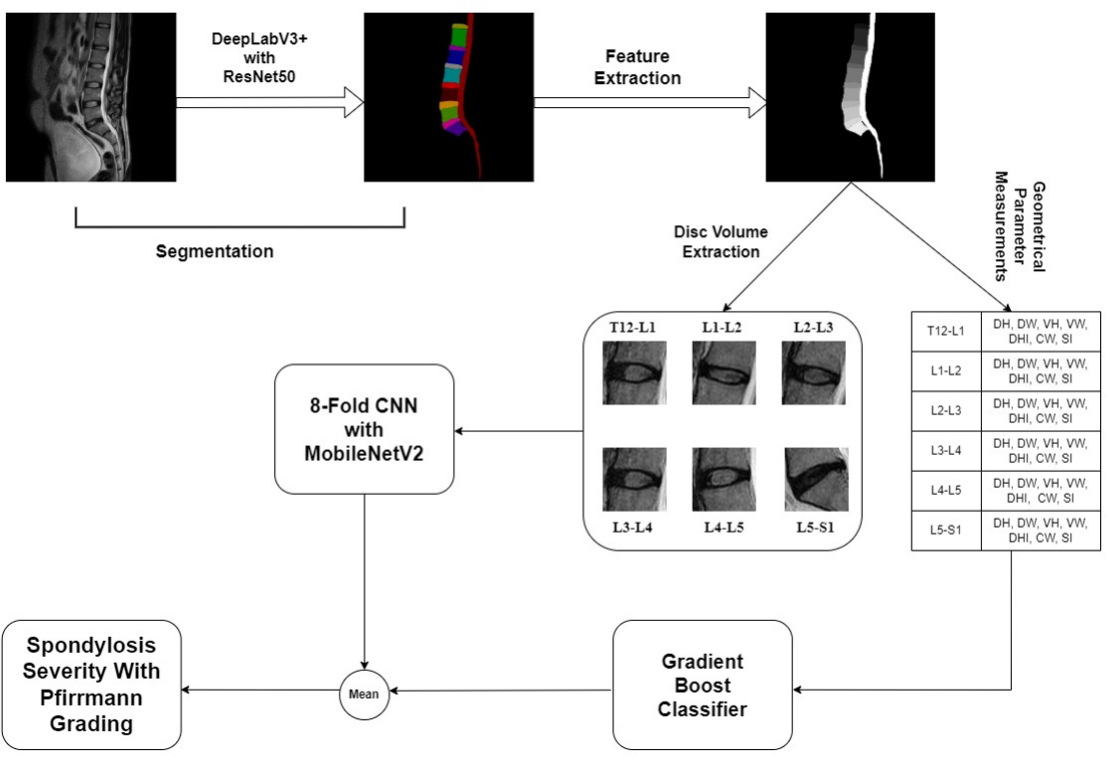
Overview of the proposed methodology in a flowchart form. CNN: convolutional neural network.

This was a prospective observational study. This paper presented an integrated methodology that leveraged AI, clinical intelligence, and radiomics to develop a predictive model, “SPOILS,” for individualized treatment planning in patients with LBP. The core objective was to assess lumbar spondylosis severity using Pfirrmann grading [8] derived from lumbar spine MRI and to guide clinical decisions toward either surgical or conservative intervention based on the predicted severity. The study analyzed 402 patients’ midsagittal T2-weighted MRIs for the segmentation model and 4260 disk slices with corresponding radiological geometric parameters for spondylosis grading. The data were collected from patients of the Neurosurgery Department at AIIMS Raebareli, covering the period from March 2023 to February 2024, as the study was approved as a time-bound project. Patients with LBP without prior surgery for back pain were included in our study. The MRIs were acquired with a resolution of 512×512 pixels. This resolution was optimal for capturing the detailed anatomical structures of the lumbar spine necessary for accurate segmentation. Image normalization, resizing, cropping, and data augmentation steps were performed during data preprocessing to increase the dataset size and ensure equal distribution of the dataset across classes, thereby avoiding overfitting. The proposed workflow consisted of several sequential steps, each designed to build upon the previous one, ensuring a systematic and efficient process from initial segmentation to the final prediction model. The workflow is depicted in Figure 1.

The first step in the workflow involved the segmentation of lumbar spine MR images. This was achieved using a transfer learning approach in a U-Net convolutional neural network (CNN) [9], which was specifically trained for multiclass segmentation. The model segmented the MR images into 14 distinct areas, including the 5 lumbar vertebrae, 1 sacrum vertebra, 6 intervertebral disks (IVDs) from T12-L1 to L5-S1, the spinal canal, and the background.

Following segmentation, the next step was to measure various geometrical parameters of the segmented structures [10]. These parameters include dimensions such as disk height, disk width, vertebrae height, vertebrae width, disk height index, canal width, and signal intensity of the IVDs, as well as other relevant metrics such as the spinal canal dimensions. The segmentation results are then used to extract the disk volume (disk slices) of the IVDs. This involved integrating the segmented disk areas across the slices of the MR images. Subsequently, the geometrical parameters and disk volumes were compiled into a structured dataset. Each entry in the dataset represented a specific slice of the IVD image and included all the extracted measurements. Additionally, the dataset was labeled with relevant Pfirrmann grading and spondylosis severity. The next step involved training a predictive model using these labeled datasets. This model was designed to predict lumbar spondylosis with Pfirrmann grading based on the extracted geometrical parameters and disk volumes. The final step toward the prediction comprised a combination of an 8-fold cross-validated CNN with MobileNetV2 [11] and a Gradient Boosting Classifier for the prediction. Unlike conventional studies that end at prediction, the novelty of our approach lay in translating these AI-derived severity assessments into tangible clinical decision mapping. By incorporating predefined thresholds and consultation with spine specialists, the model outcomes were used to recommend individualized treatment pathways—either conservative (medical or physiotherapy based) or surgical intervention. This AI–clinical intelligence and radiomics fusion facilitated a more objective, reproducible, and scalable framework for spine care, potentially reducing interobserver variability and optimizing therapeutic strategies.

### Dataset

The dataset used in this study comprised midsagittal T2-weighted lumbar spine MR images. The study was funded by the Department of Health Research–Multi-Disciplinary Research Units and started after institutional ethical clearance in collaboration with AIIMS Raebareli and National Institute of Technology Meghalaya.

### Data Segmentation and Annotation

**Figure 2.**
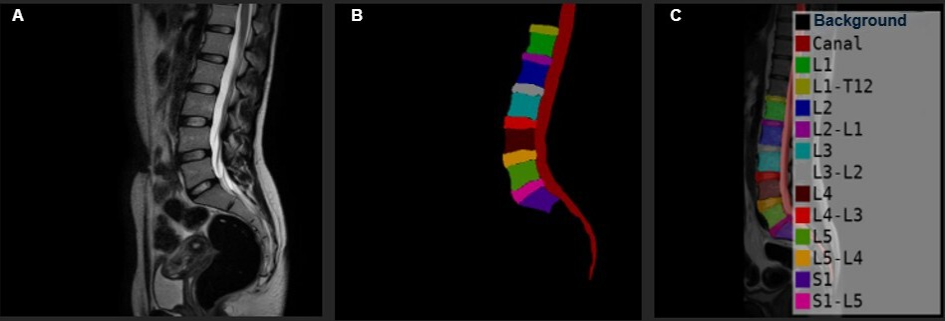
Illustration of a labeled dataset for segmentation: (A) original magnetic resonance imaging (MRI) scan, (B) expert-annotated ground truth mask, and (C) corresponding class names for each region in the mask. L1 to L5: lumbar vertebrae; T12-L1 and L5-S1: intervertebral disks; S1: sacral vertebra.

The MR images were randomly divided into training and testing sets after they were exported. Random allocation ensured that the training and testing sets were representative of the entire dataset, thereby strengthening the model’s robustness. Developing efficient semantic segmentation models for medical imaging required precise data segmentation and labeling. LabelMe (version 3.3.6; MIT Computer Science and Artificial Intelligence Laboratory) [12], a popular image annotation program that enabled accurate and effective labeling, was used to tag all the MRIs to aid semantic segmentation. This procedure included precisely marking 14 different regions necessary for a thorough study of the lumbar spine. The 14 regions comprised 1 sacral vertebra (S1), 5 lumbar vertebrae (L1 to L5), 6 IVDs (T12-L1 to L5-S1), spinal canal, and background as shown in (Figure 2).

### Segmentation Models

**Figure 3.**
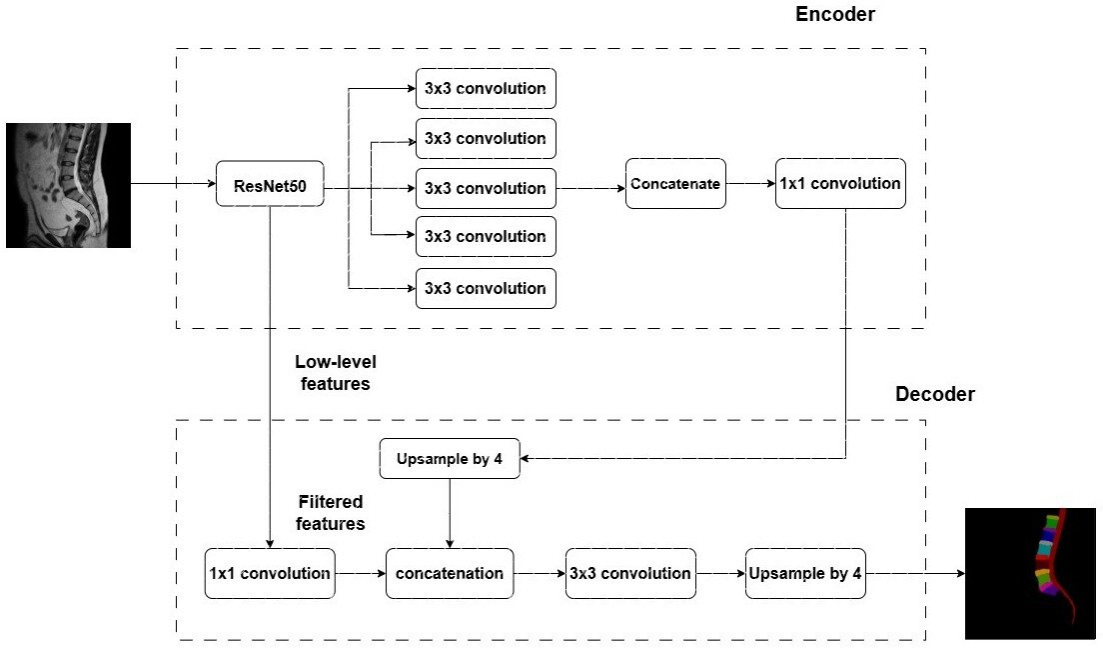
Architecture of the proposed model.

For accurate and precise multiclass segmentation of lumbar MR images, we used the DeepLabV3+ [13] model with ResNet50 [14] as the encoder. DeepLabV3+ enhances the original DeepLabV3 by incorporating a more advanced decoder module, making it a state-of-the-art semantic segmentation model. It leveraged a robust CNN backbone to extract high-level features from input images, with ResNet50 being the chosen architecture for the encoder. ResNet50, known for its depth and ability to capture intricate features and semantics in images, was pretrained on the ImageNet dataset, which further enhanced its performance in medical image segmentation tasks (Figure 3).

The architecture of the DeepLabV3+ model begins with an input layer of shape (512, 512, 3), suitable for processing the 512×512 MR images. The core component of this architecture was the Atrous Spatial Pyramid Pooling (ASPP) module, which was crucial for efficient multiscale data aggregation. The ASPP module consisted of parallel atrous convolutional layers with varying dilation rates, allowing it to capture context information at multiple scales. The use of dilated convolutions in the ASPP module expanded the receptive field of each convolutional layer, enabling the model to gather both local and global context information effectively. Additionally, the ASPP module included a 1×1 convolutional layer to reduce the dimensionality of the feature maps and an average pooling layer to gather global context information. Following the ASPP module, the decoder module integrated features from the encoder with upsampled feature maps to produce dense pixel-wise predictions. This module used skip connections from the encoder and comprised multiple convolutional and upsampling layers. By concatenating features from different spatial scales, the decoder refines the predictions, thereby improving segmentation accuracy. In practice, the decoder module first increased the spatial resolution of the feature maps through bilinear upsampling and then combined these upsampled maps with features extracted by the encoder. The output layer of this architecture included a final convolutional layer and a softmax activation function, which generated the segmentation masks.

This sophisticated approach enabled the DeepLabV3+ model to deliver precise and detailed segmentations, making it highly effective for the segmentation of lumbar spine MR images. The combination of ResNet50’s powerful feature extraction capabilities and the ASPP module’s multiscale context aggregation ensured high accuracy in delineating anatomical structures in medical images.

### Study Parameters: Geometrical and Disk Volume Data

In this section, we describe the process of extracting geometrical data [10] from the segmented image dataset to predict lumbar spondylosis. The disk volume extraction process involved several stages, including bounding volume estimation, volume resizing, and normalization. Bounding volume estimation was achieved by detecting and sorting the corner points of the segmented IVDs. Once the bounding volumes were estimated, the next step was to resize the extracted volumes to a standardized dimension. Resizing ensures that all IVDs were represented consistently, regardless of variations in patient anatomy or image acquisition parameters. During this step, the extracted disk volumes were adjusted to fit a uniform scale while maintaining the aspect ratio to preserve the anatomical integrity of the IVDs. After resizing, disk volume normalization was performed by adjusting the intensity values of the extracted volumes. By normalizing the intensity values, we ensured that the signal intensities of the disks were comparable across the entire dataset.

The extracted geometrical parameters included average disk height, central disk height (height at the midpoint of the IVDs), vertebrae height, average disk width, midpoint disk width, disk height index, signal intensity, and signal intensity difference. To calculate these geometrical parameters, we used the method described by Kumar et al [10]. The extracted geometrical parameters and signal intensity measurements were then compiled into a structured dataset. Each entry in this dataset represented a specific slice of an IVD image and included all the extracted measurements. Additionally, the dataset was labeled with relevant Pfirrmann grading, and spondylosis severity was categorized as low, medium, or high pain, providing a comprehensive dataset for further analysis and predictive modeling. This structured dataset, containing detailed geometric and signal intensity information along with spondylosis grades, formed the foundation for developing predictive models. These models aimed to improve the diagnosis and treatment planning for lumbar spine spondylosis, ultimately contributing to better patient care and outcomes.

### Labeling

Each extracted disk volume and geometrical dataset was then labeled according to an 11-class scheme. This scheme incorporated the Pfirrmann grading system, which classified disk degeneration into 5 grades based on structural and compositional changes observed in MR images. The Pfirrmann grades ranged from grade 1 (healthy disk) to grade 5 (severely degenerated disk). Additionally, the dataset included a spondylosis severity index, which assessed the presence and extent of osteophytes, disk space narrowing, and other degenerative changes. The spondylosis severity was categorized into mild, moderate, and severe. Both the Pfirrmann grades and spondylosis severity were combined to form classes for labeling. The dataset contained a total of 11 classes, including 1L, 2L, 3L, 3M, 3H, 4L, 4M, 4H, 5L, 5M, and 5H. L, M, and H represented low, moderate, and high severity, respectively.

### Prediction Model for Spondylosis

To predict Pfirrmann grading and spondylosis severity using the extracted disk volume dataset, we used and compared the performance of 3 different models: a CNN, a CNN with MobileNetV2, and a CNN with VGG16. The models were evaluated based on accuracy and intersection over union (IoU) metrics over 150 epochs. Similarly, for the geometrical dataset, we evaluated 4 different models: logistic regression, XGBClassifier, Gradient Boosting Classifier, and support vector machine classifier.

### Clinical Decision Support Integration

The novelty of the SPOILS model lies in its integration of AI-derived severity classification with patient-reported outcomes—Oswestry Disability Index (ODI) and Numerical Rating Scale (NRS) for pain—to inform treatment pathways. On the basis of the predicted class and clinical scores, patients were triaged into the following decision strata:

- Surgical intervention candidates—patients classified as 3H, 4M, 4H, 5M, or 5H with high ODI or NRS scores were directed toward surgical consultation.
- Interventional pain management candidates—patients with 3M, 4L, or 5L were recommended for transforaminal epidural steroid injections, with surgery considered if pain persists despite intervention.
- Conservative management candidates—patients with lower severity classes (1L, 2L, or 3L) and mild to moderate ODI or NRS scores were advised to undergo pharmacological therapy and physiotherapy. Transforaminal steroid injections were considered if symptoms remained refractory to conservative treatment.

## Results

### Demographic Profile

A total of 402 patients underwent lumbar spine MRI using a 1.5-T MRI unit. Out of these 402 patients, 55 (13.7%) were treated with transforaminal nerve block, 40 (10%) were surgically managed, and the remaining patients (n=307, 76.4%) were managed with medicines. The dataset contained MR images from both male and female patients. The description of the dataset is provided in Table 1.

**Table 1.** Gender-wise dataset description^a^.

Sex	Patients (n=402), n (%)	Magnetic resonance images (n=1460), n (%)
Male	235 (58.5)	855 (58.6)
Female	167 (41.5)	605 (41.4)

a All data were obtained from the Department of Neurosurgery, All India Institute of Medical Sciences, Raebareli, Uttar Pradesh, India.

This helped us in understanding the role of sex in changes in geometrical parameters. In Table 2, data are presented according to age group. This helped us understand the effect of age on geometric parameters. This helped in understanding the aging factors involved in the changes of geometrical aspects.

**Table 2.** Age group-wise dataset description^a^.

Age group (years)	Patients (n=402), n (%)	Magnetic resonance images (n=1460), n (%)
0‐15	10 (2.5)	40 (2.7)
16‐30	92 (22.9)	370 (25.3)
31‐45	105 (26.1)	410 (28.1)
46‐60	102 (25.4)	385 (26.4)
>60	93 (23.1)	255 (17.5)

a All data were obtained from the Department of Neurosurgery, All India Institute of Medical Sciences, Raebareli, Uttar Pradesh, India.

### Study Parameters

The results of the segmentation model using DeepLabV3+ with ResNet50 as the encoder demonstrated a high level of performance in the segmentation of lumbar spine MR images. Initially, the model achieved an accuracy of 95.5%, a precision of 92.8%, a recall of 93.2%, a Dice coefficient of 93%, and an IoU of 87.1%. These metrics indicated the model’s strong capability in accurately identifying and delineating the various anatomical structures within the lumbar spine MR images.

**Figure 4.**
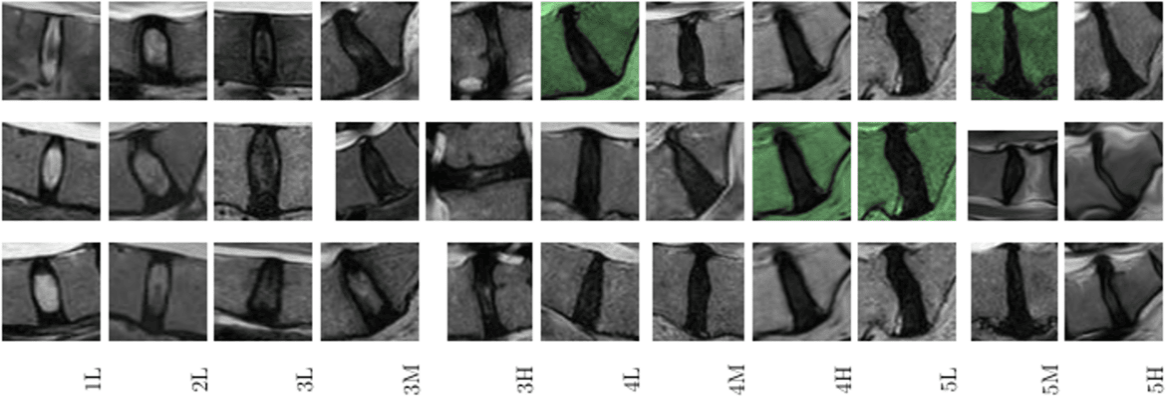
Example illustrating Pfirrmann grading–based spondylosis severity labeling of intervertebral disk degeneration.

To further validate and potentially enhance the performance of the segmentation model, we implemented an 8-fold cross-validation technique. Cross-validation is a robust method for assessing the generalizability and reliability of a model by partitioning the dataset into multiple folds and ensuring that each fold serves as a validation set at least once. Through this rigorous validation approach, the model’s performance metrics significantly improved. After applying 8-fold cross-validation, the segmentation model exhibited an impressive increase in performance, achieving an accuracy of 98.7%, a precision of 96.95%, a recall of 97.1%, a Dice coefficient of 96.9%, and an IoU of 94.8%. These enhanced results underscored the model’s robustness and its ability to consistently produce accurate and precise segmentations across different subsets of the data. Figures4  5 illustrate representative segmentation outputs obtained using the proposed models.

**Figure 5.**
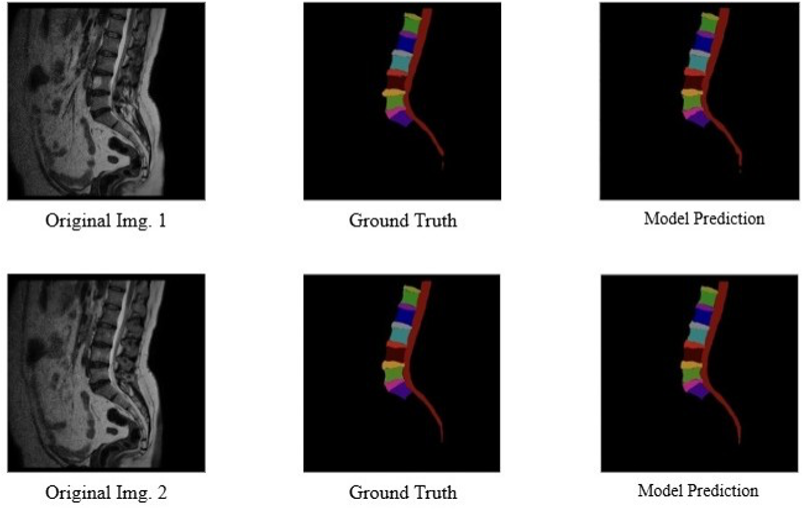
Results of segmentation predicted by the models on sample inputs.

**Figure 6.**
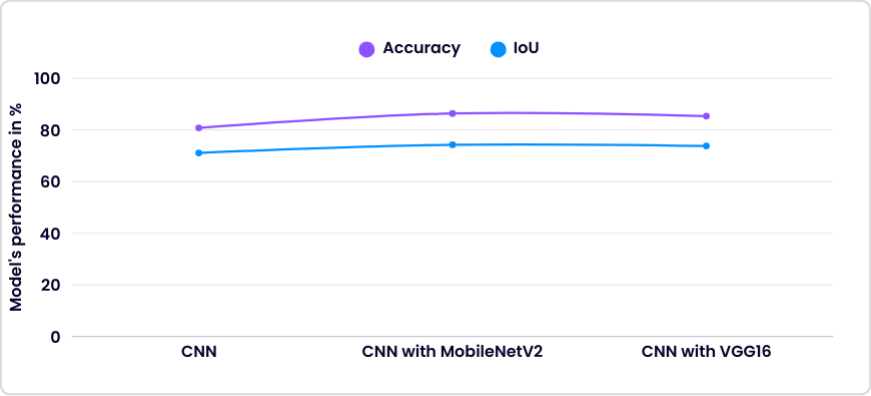
Performance of prediction model over the extracted disk volume dataset. CNN: convolutional neural network; IoU: intersection over union.

The results of the prediction model for spondylosis over the extracted disk volume dataset are depicted in Figure 6.

Among these models, the CNN with MobileNetV2 demonstrated superior performance (accuracy=86.40% and IoU=74.27%) compared with the other 2 models. To further enhance this model’s robustness and generalizability, we used an 8-fold cross-validation technique. This method involved partitioning the dataset into 8 subsets, training the model on 7 subsets, and validating it on the remaining subset. This process was repeated 8 times, with each subset serving as the validation set once. The implementation of 8-fold cross-validation significantly improved the performance of the CNN with MobileNetV2. After cross-validation, the model’s accuracy increased to 97.84%, and the IoU improved to 96.76%. These results indicated that the CNN with MobileNetV2, enhanced by cross-validation, provided a highly accurate and reliable method for predicting Pfirrmann grading and spondylosis severity.

**Figure 7.**
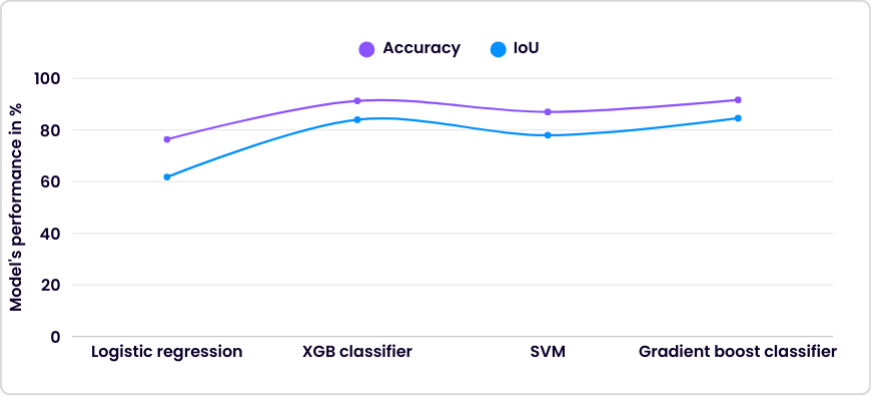
Performance of prediction model over the extracted geometrical dataset. IoU: intersection over union; SVM: support vector machine; XGB: Extreme Gradient Boosting.

Figure 7 shows the results of the prediction model for spondylosis using the geometrical dataset. Among these models, the Gradient Boosting Classifier demonstrated superior performance (accuracy=91.65% and IoU=84.59%) compared with the other models.

Our final model for predicting Pfirrmann grading and spondylosis severity leveraged an ensemble approach that combined the strengths of 2 distinct models: an 8-fold cross-validated CNN with MobileNetV2 and a Gradient Boosting Classifier. The predictions from both models are then combined by taking the mean of their outputs, leading to a final, more robust prediction of spondylosis severity and Pfirrmann grading.

## Discussion

### Principal Findings

In this study, we developed and evaluated a comprehensive framework for predicting Pfirrmann grading and spondylosis severity using both MRI-based deep learning models and radiological geometrical parameters [10]. The results demonstrated that combining image-based representations with quantitative anatomical features could provide robust and reliable predictions, supporting the feasibility of a fully automated decision support system for lumbar spine degeneration assessment.

Our experiments with image-based models showed that deep convolutional neural networks were well suited for learning discriminative features related to IVD degeneration. Among the evaluated architectures, CNN integrated with MobileNetV2 consistently outperformed the baseline CNN and CNN-VGG16 models, achieving an accuracy of 86.4% and an IoU of 74.27%. The superior performance of MobileNetV2 could be attributed to its depthwise separable convolutions and efficient feature reuse, which allowed the network to capture fine-grained textural and structural variations in disk morphology while maintaining computational efficiency. Further performance gains were achieved through 8-fold cross-validation, resulting in an accuracy of 97.84% and an IoU of 96.76%. This substantial improvement highlighted the critical role of cross-validation in reducing overfitting, improving generalization, and producing more reliable performance estimates on unseen data—an especially important consideration in medical imaging studies with limited datasets.

These findings align with a growing body of literature demonstrating the effectiveness of CNN-based approaches for automated Pfirrmann grading. Several recent studies have reported CNN performance approaching or even matching expert-level assessments, reinforcing the clinical potential of deep learning for disk degeneration analysis. Previous work by Nikpasand et al [15] also demonstrated strong agreement between CNN-predicted Pfirrmann grades and expert annotations, surpassing interobserver agreement among human graders (78% agreement; Fleiss κ=0.68). Their findings supported the notion that automated systems could reduce subjective variability and provide more consistent grading than manual assessment alone. Our results further extended this observation by demonstrating that careful model selection, validation strategies, and dataset construction could lead to substantial gains in predictive reliability.

In contrast, some studies have reported exceptionally high classification accuracies exceeding 95% across all Pfirrmann grades [16,17]. Griffith et al [18] evaluated a comprehensive framework for modified Pfirrmann grading. Our attempts to replicate similar performance using comparable architectures and dataset sizes resulted in comparable accuracies ranging from 81% to 86% for Pfirrmann grading, with spondylosis severity depending on the lumbar level.

Beyond image-based learning, this study also explored predictive modeling using radiological geometrical parameters derived from segmented disk volumes. Among the evaluated classical machine learning models, the Gradient Boosting Classifier achieved the best performance, with an accuracy of 91.65% and an IoU of 84.59%. Although slightly lower than the best-performing CNN, this result demonstrated the strong predictive value of quantitative anatomical features. Gradient Boosting’s ability to iteratively correct errors made by weak learners made it particularly effective for capturing nonlinear relationships within structured numerical data. These findings suggest that geometric measurements provide complementary information to image-based features and should not be overlooked in automated spinal analysis.

Finally, combining the predictions from the CNN with MobileNetV2 and the Gradient Boosting Classifier resulted in consistent and reliable estimates of Pfirrmann grading and spondylosis severity. This hybrid strategy leveraged the strengths of deep learning in capturing complex visual patterns and the interpretability and robustness of feature-based machine learning models. Such an ensemble approach is particularly attractive in clinical contexts, where both accuracy and reliability are critical.

Overall, the findings of this study reinforced the potential of automated, multimodal frameworks for lumbar spine assessment. By emphasizing robust validation, transparent methodology, and the integration of complementary data representations, this work contributed toward the development of clinically deployable tools that could support radiologists and spine surgeons in objective grading and treatment planning for patients with spondylosis.

### Decision-Making

We developed an end-to-end application “SPOILS” that leveraged predicted classes alongside clinical scores, such as the ODI and the NRS for pain, to support personalized treatment planning for patients with LBP. This AI-driven stratification system enabled a tailored, data-informed approach that minimized subjectivity in clinical decision-making. By integrating predictive analytics with validated clinical measures, the platform enhanced treatment precision and consistency, ultimately improving patient outcomes and offering a scalable solution for effective LBP management.

Previous studies have highlighted the potential of machine learning in medical imaging for various applications, including the detection of spinal abnormalities and the prediction of disease progression [19-29]. However, there remains a need for robust, clinically validated systems specifically designed for lumbar spine spondylosis, as well as complete integration of model outputs into clinical workflows. This research sought to fill this gap by developing an end-to-end pipeline and validating predictive models that could accurately predict Pfirrmann and spondylosis grades, thereby supporting clinical decision-making regarding medical or surgical interventions. By combining deep learning models with structured clinical logic, this system aligned with real-world needs in spine care, particularly in settings where access to subspecialist expertise may be limited.

### Limitations and Future Work

Despite the promising results, there were limitations to our study. The dataset, while comprehensive, was still limited in size, and further validation on larger, more diverse datasets is necessary to confirm the generalizability of our model. Additionally, while the ensemble approach improved accuracy, it also increased the complexity of the model.

Future work will focus on expanding the dataset, incorporating additional features such as patient demographics and clinical history, and exploring other ensemble techniques to further enhance model performance. Additionally, the integration of explainable AI techniques will be considered to provide greater transparency in model predictions, which is crucial for clinical adoption.

### Conclusions

Our study demonstrated the effectiveness of combining CNN with MobileNetV2 and Gradient Boosting Classifier for predicting Pfirrmann grading and spondylosis severity. The model not only achieved state-of-the-art predictive performance but also served as the backbone of an interpretable and clinically validated decision support system. This positions the model as a scalable solution for personalized LBP management, with potential applicability in tertiary hospitals and spine specialty clinics.

## Abbreviations

AI: artificial intelligence
AIIMS: All India Institute of Medical Sciences
ASPP: Atrous Spatial Pyramid Pooling
CNN: convolutional neural network
IoU: intersection over union
IVD: intervertebral disk
LBP: low back pain
MR: magnetic resonance
MRI: magnetic resonance imaging
NRS: Numerical Rating Scale
ODI: Oswestry Disability Index
SPOILS: Software to Predict Outcome in Lumbar Spondylosis

## References

[1] GBD 2017 Disease and Injury Incidence and Prevalence Collaborators (2018). Global, regional, and national incidence, prevalence, and years lived with disability for 354 diseases and injuries for 195 countries and territories, 1990–2017: a systematic analysis for the Global Burden of Disease Study 2017. Lancet.

[2] Leboeuf-Yde C, Kyvik KO (1998). At what age does low back pain become a common problem? A study of 29,424 individuals aged 12-41 years. Spine (Phila Pa 1976).

[3] Ganesan S, Acharya AS, Chauhan R, Acharya S (2017). Prevalence and risk factors for low back pain in 1,355 young adults: a cross-sectional study. Asian Spine J.

[4] Coppieters I, Meeus M, Kregel J (2016). Relations between brain alterations and clinical pain measures in chronic musculoskeletal pain: a systematic review. J Pain.

[5] Wu A, March L, Zheng X (2020). Global low back pain prevalence and years lived with disability from 1990 to 2017: estimates from the Global Burden of Disease Study 2017. Ann Transl Med.

[6] Nijs J, Apeldoorn A, Hallegraeff H (2015). Low back pain: guidelines for the clinical classification of predominant neuropathic, nociceptive, or central sensitization pain. Pain Physician.

[7] Chien JJ, Bajwa ZH (2008). What is mechanical back pain and how best to treat it?. Curr Pain Headache Rep.

[8] Pfirrmann CW, Metzdorf A, Zanetti M, Hodler J, Boos N (2001). Magnetic resonance classification of lumbar intervertebral disc degeneration. Spine (Phila Pa 1976).

[9] Ronneberger O, Fischer P, Brox T U-net: convolutional networks for biomedical image segmentation. https://link.springer.com/chapter/10.1007/978-3-319-24574-4_28.

[10] Kumar P, Singh S, Balabantaray BK, Nayak R (2026). Automated quantitative analysis of the lumbar spine: a comprehensive approach. J Imaging Inform Med.

[11] Sandler M, Howard A, Zhu M, Zhmoginov A, Chen LC MobileNetV2: inverted residuals and linear bottlenecks.

[12] Russell BC, Torralba A, Murphy KP, Freeman WT (2007). LabelMe: a database and web-based tool for image annotation. Int J Comput Vis.

[13] Chen LC, Zhu Y, Papandreou G, Schroff F, Adam H Encoder-decoder with atrous separable convolution for semantic image segmentation.

[14] He K, Zhang X, Ren S, Sun J Deep residual learning for image recognition.

[15] Nikpasand M, Middendorf JM, Ella VA (2024). Automated magnetic resonance imaging-based grading of the lumbar intervertebral disc and facet joints. JOR Spine.

[16] Liawrungrueang W, Cholamjiak W, Sarasombath P, Jitpakdee K, Kotheeranurak V (2024). Artificial intelligence classification for detecting and grading lumbar intervertebral disc degeneration. Spine Surg Relat Res.

[17] Baur D, Bieck R, Berger J (2025). Automated three-dimensional imaging and Pfirrmann classification of intervertebral disc using a graphical neural network in sagittal magnetic resonance imaging of the lumbar spine. J Imaging Inform Med.

[18] Griffith JF, Wang YX, Antonio GE (2007). Modified Pfirrmann grading system for lumbar intervertebral disc degeneration. Spine (Phila Pa 1976).

[19] Bishop JB, Szpalski M, Ananthraman SK, McIntyre DR, Pope MH (1997). Classification of low back pain from dynamic motion characteristics using an artificial neural network. Spine (Phila Pa 1986).

[20] Jaremko JL, Poncet P, Ronsky J (2001). Estimation of spinal deformity in scoliosis from torso surface cross sections. Spine (Phila Pa 1986).

[21] Liszka-Hackzell JJ, Martin DP (2002). Categorization and analysis of pain and activity in patients with low back pain using a neural network technique. J Med Syst.

[22] Veronezi CC, de Azevedo Simões PW, Dos Santos RL (2011). Computational analysis based on artificial neural networks for aiding in diagnosing osteoarthritis of the lumbar spine. Rev Bras Ortop.

[23] Wang J, Fang Z, Lang N, Yuan H, Su MY, Baldi P (2017). A multi-resolution approach for spinal metastasis detection using deep Siamese neural networks. Comput Biol Med.

[24] Kim JS, Merrill RK, Arvind V (2018). Examining the ability of artificial neural networks machine learning models to accurately predict complications following posterior lumbar spine fusion. Spine (Phila Pa 1986).

[25] Muhaimil A, Pendem S, Sampathilla N (2024). Role of artificial intelligence model in prediction of low back pain using T2 weighted MRI of lumbar spine. F1000Res.

[26] Zhang J, Li H, Lv L, Zhang Y (2017). Computer-aided Cobb measurement based on automatic detection of vertebral slopes using deep neural network. Int J Biomed Imaging.

[27] Berg B, Gorosito MA, Fjeld O (2024). Machine learning models for predicting disability and pain following lumbar disc herniation surgery. JAMA Netw Open.

[28] Stanley RJ, Long R (2001). A radius of curvature-based approach to cervical spine vertebra image analysis. Biomed Sci Instrum.

[29] Bhak Y, Ahn TK, Peterson TA, Han HW, Nam SM (2024). Machine learning models for low back pain detection and factor identification: insights from a 6-year nationwide survey. J Pain.

